# High-Throughput Toxicity Screening with *C. elegans*: Current Platforms, Key Advantages, and Future Directions

**DOI:** 10.1021/acs.est.5c12562

**Published:** 2026-01-08

**Authors:** Timothy A. Crombie, Tobias Pamminger, Erik C. Andersen, Scott Glaberman

**Affiliations:** a Department of Biomedical Engineering and Science, 5401 Florida Institute of Technology, Melbourne, Florida 32901, United States; b 39092 Bayer AG, CropScience Division, Monheim 40789, Germany; c Department of Biology, Johns Hopkins University, Baltimore, Maryland 21218, United States; d Centre for Environmental Research and Justice, School of Biosciences, 1724 University of Birmingham, Birmingham B15 2TT, United Kingdom

**Keywords:** *Caenorhabditis elegans*, human health, risk assessment, One Health, toxicity testing, high-throughput assays

## Abstract

Nematodes, including the model *Caenorhabditis elegans*, pose many advantages for high-throughput screening (HTS) of chemical toxicity, such as ease of culture, short life cycles, low maintenance costs, and a wide array of available strains and mutants. Several HTS platforms have already been developed to rapidly assess multiple endpoints, including behavior, growth, and reproduction of *C. elegans*. Here, we summarize the available methodologies for HTS in C. elegans and evaluate their strengths and limitations for routine chemical screening. We also assess the relationship between *C. elegans* HTS data and toxicity information from other common surrogate species, including fish, invertebrates, and algae, as well as data from other HTS assays. Notably, image-based HTS data yielded strong concordance between toxicological endpoints for *C. elegans* and established ecotoxicological surrogates. Finally, we make recommendations for how to improve existing platforms and where collaboration and investment are needed to make nematodes an integral part of the battery of alternatives to reduce vertebrate testing.

## Graphical abstract

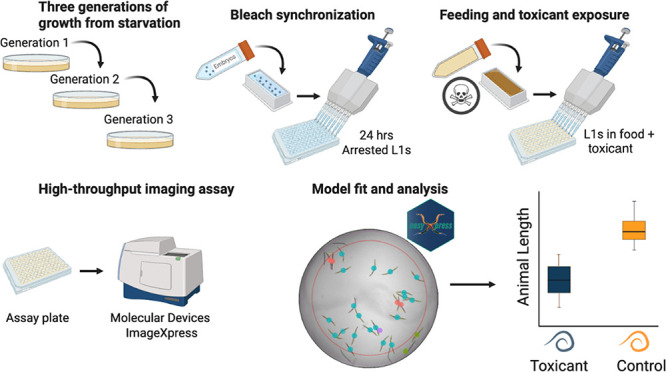

## Introduction

Environmental toxicology is undergoing a major transformation, driven by global efforts to reduce vertebrate animal testing and improve animal welfare.^1^ At the same time, advances in molecular and computational toxicology are expanding our ability to evaluate chemical impacts on humans and ecosystems. One major area of progress is the use of high-throughput screening (HTS) to rapidly assess toxicity. Although emerging cell-based systems and organoids show promise,^2,3^ HTS using whole organisms remains critical because they integrate chemical stressors across developmental, physiological, and behavioral pathways in ways that isolated cells cannot.^4^ In addition, certain toxicological phenomena (*e.g*., toxic metabolites) can often only be detected in intact organisms that have both the metabolically active tissue and the molecular target present.^5^ However, few species are used for *in vivo* HTS,^6,7^ especially with the intention to be used within a regulatory chemical risk assessment framework. Moreover, most current HTS models are aquatic, raising concerns about missing toxicological impacts on terrestrial species. Here, we evaluate nematodes as a model system for HTS in environmental toxicology with a particular focus on *Caenorhabditis elegans*.

Nematodes offer multiple advantages for toxicity testing and environmental risk assessment.^8^ They are relatively easy to culture in the lab, requiring minimal space and resources, and are supported by an extensive research community. Nematodes can also be cryopreserved and stored indefinitely, facilitating long-term studies and experimental reproducibility. Many of the latest advances in molecular biology have been established in *C. elegans*, including RNAi,^9^ CRISPR-Cas9 genome editing,^10^ and several published genomes are available for both *C. elegans* and other nematodes.^11–16^ Thousands of wild strains and mutants exist for testing specific mechanistic hypotheses related to chemical toxicity and modes of action.^17^

The established role of *C. elegans* as a model for developmental biology makes it potentially useful for investigating toxicity mechanisms and effects relevant to human, animal, and environmental health. Most core developmental signaling pathways are conserved between *C. elegans* and humans,^18–20^ and the disruptive effects of mammalian developmental toxicants on *C. elegans* are well established.^21–25^ Moreover, *C. elegans* neurobiology is exceptionally characterized, and both its neurotransmitter systems and neuronal cell fate specification pathways are largely conserved with mammals.^26,27^ These parallels in neurobiology likely contribute to the high concordance between neuroactive toxicants in *C. elegans* and mammals.^28^ For example, *C. elegans* recapitulates key mammalian features of metal-induced neurotoxicity, including oxidative stress, dopaminergic neurodegeneration, and motor deficits.^27^ Nematodes, such as *C. elegans*, also play vital roles in terrestrial ecosystem functioning,^29,30^ including decomposing plant material,^31^ and shaping microbial diversity.^32^ Far fewer standardized toxicity testing guidelines exist for terrestrial organisms compared to their aquatic counterparts.^33^ Taken together, the molecular and developmental strengths of *C. elegans*, along with its ecological relevance, make it well suited to assess potential human and ecological impacts of chemicals under a One Health framework that aims to integrate the health of people, animals, and ecosystems.^34^

Many reviews have already explored the advantages of *C. elegans* as a model for basic and applied toxicology, including its genetic tractability, use in mechanistic studies, alignment with key toxicological pathways, and use to assess environmental contaminants within a regulatory context.^18,35–39^ These publications have also catalogued the broad range of applications and toxicological endpoints available in *C. elegans*, from growth and reproduction to neurotoxicity and oxidative stress, and have outlined its potential for both biomedical and environmental applications. Rather than reiterating these general advantages, we focus here on the diverse HTS platforms developed for *C. elegans*, which are critical to its future role in predictive toxicology and risk assessment.

Several HTS platforms have already been optimized for *C. elegans*, providing a strong foundation for future testing frameworks. Microfluidic technologies employ intricate on-chip control layers and chamber arrays, significantly augmenting experimental control and enabling more refined analysis in genetic and chemical screens.^40,41^ Other HTS systems capture behavioral phenotypes in multiwell formats, enabling analysis of neurotoxic or sublethal effects at previously unprecedented scale.^42–44^ HTS platforms based on large-particle flow-based (LPFB) systems have been optimized to rapidly quantify the size of individual nematodes across different developmental stages, providing a measure of growth for animals exposed to environmental toxicants.^8,45–52^ Finally, microscopy-based imaging platforms have been used to measure growth by conducting chemical exposures in multiwell plates and then using analytical software to measure chemical effects at the individual level.^53–55^

Despite their promise, the regulatory relevance of nematode HTS systems for hazard and risk assessment remains poorly defined. It is especially unclear how *C. elegans* or other nematodes could represent, or even predict, chemical effects observed in other standard *in vivo* and *in vitro* surrogate testing systems. Critically, the concept of using cross-species comparisons to inform ecological and human risk assessment is established and broadly accepted within the context of evolutionary toxicology.^56–61^ An important outgrowth of this field is the adverse outcome pathway (AOP) framework, which provides a structured way to link molecular initiating events and key biological processes associated with chemical susceptibility across taxa.^62^ By capturing mechanistic steps that are often functionally conserved among species, the AOP framework offers a basis for predicting when toxicant effects in models such as *C. elegans* could translate to other organisms.^60^ Accordingly, when the key biological events within relevant AOPs are conserved, species that share these pathways are expected to show similar patterns of sensitivity across chemicals. A previous review described the use of the *C. elegans* model for predictive toxicology, but at the time, most available studies tested a small number of compounds.^63^ One study included in the review did show comparable rank ordering of toxicity between *C. elegans* LPFB data and zebrafish embryo tests for hundreds of compounds,^47^ but a systematic assessment of the suitability of nematodes for representing a broader range of taxa in the context of chemical assessment has yet to be performed.

Here, we attempt to fill this critical knowledge gap and outline a strategic plan to advance nematode-based HTS for environmental risk assessment. We first compared methodologies across the major HTS platforms. We then summarize available data on toxicity variation among *C. elegans* strains within HTS frameworks. Next, we compare *C. elegans* HTS data to other ecological and human health surrogates, including rodents, fish, aquatic invertebrates, and algae. Finally, we discuss key knowledge gaps and propose future research directions for expanding and improving nematode HTS systems. Our aim is to pinpoint the strengths and limitations of HTS screening in nematodes and identify where further investment is needed. By systematically comparing *C. elegans* HTS data with those of other established *in vivo* and *in vitro* models, we aim to better assess the relevance of nematode testing within the broader spectrum of toxicological research. All data and code required to reproduce our analyses are available in the associated GitHub repository (https://github.com/Crombie-Lab/nematode-hts-toxicology), and the harmonized cross-species HTS data are provided in the Supporting Information.

## Comparison of HTS Platforms

Over the last several decades, four major HTS systems have emerged to quantify toxicologically relevant phenotypes in C*. elegans* and related species. Each platform represents a unique balance of experimental control, scalability, phenotypic resolution, and cost (Table 1).

**Table 1:** Comparison of Established High-Throughput Screening (HTS) Systems for *C. elegans* and Related Species^a^

category	microfluidics	behavioral	large-particle flow-based	imaging-based
**platform description**	employs microscale channels to manipulate and analyze nematodes	uses megapixel camera arrays and extracts behavioral traits from videos	uses flow cytometry and sorting based on length, density, fluorescence	uses image capture and analysis algorithms
**setup cost and** **effort**	high	low	high	low
**operating cost**	high	low	high	moderate
**endpoints captured**	morphology, behavior, biochemical assays	behavior	length, optical density, fluorescence intensity	morphology, behavior, fluorescence
**data processing effort**	moderate: segment nematodes from images (no untangling)	very High: track segmented nematodes across videos (untangling)	low: Process optical values for nematodes	moderate–high: segment nematodes from images (untangling)
**data storage requirements**	moderate: raw images	high: raw video	low: text-based files of nematode values	moderate: raw images
**throughput scale**	low: depends on device design and flow rate, tens of animals per hour	Mmoderate: modular scaling, can be thousands of nematodes per hour	high: capable of analyzing tens of thousands of nematodes per hour	high: depends on imaging speed, can be tens of thousands of nematodes per hour
**life-stages tested**	larval stages and adults	larval stages and adults	various, including larvae, adults, and embryos	various, including larvae, adults, and embryos

a Untangling refers to the computation separation of overlapping or intersecting nematode bodies from images.

### Microfluidic Systems

Microfluidics technology offers multiple advantages for nematode HTS by facilitating precise environmental control and efficient nematode handling and enabling experiments that are challenging or impossible with traditional methods. This technology leverages fluid flows at the micron scale to create predictable and controllable conditions such as accurate flow rates, concentration gradients, and shear rates, with the added benefit of requiring minimal sample volumes.^64,65^ Microfluidic devices are primarily fabricated from polydimethylsiloxane (PDMS), a flexible, optically transparent, and biocompatible material, using soft lithography techniques, making device production accessible, rapid, and cost-effective.^41^ These devices have significantly advanced research areas like behavioral analysis, high-resolution imaging, and optogenetics by allowing precise manipulation and observation of *C. elegans* in diverse experimental setups.^66–70^

The benefits of microfluidics in *C. elegans* research include the ability to measure toxicant effects where high-resolution microscopy is required.^71^ This advance is made possible by the technology’s capability to immobilize animals without anesthesia, monitor their behavior in response to various stimuli, and perform detailed imaging at cellular or subcellular levels.^72,73^ The imaging capabilities of microfluidic devices can be augmented with acoustofluidics, which enable fine rotational control of *C. elegans* within the device, improving visualization of internal structures.^74^ Microfluidic platforms have also been developed to assess chemical effects on embryo viability.^75^ The data output from these platforms can inform chemical hazard characterization, ranging from behavioral responses to fine-scale morphological and functional changes at the cellular level.^76–78^

However, the use of microfluidics in chemical screening, although promising, also presents challenges, particularly in achieving truly high-throughput solutions. Thus far, most applications operate at throughputs several orders of magnitude lower than the flow-based and imaging platforms discussed below, with some recent exceptions.^73^ Additionally, application-specific device development is often required to expand the range of possible experiments, ensure biological relevance within microfluidic environments, and address any potential effects of microfluidic materials on test organisms.^65,79^ As the field progresses, the design and functionality of microfluidic devices are expected to evolve, enhancing their use and broadening their applicability in biological research, especially toward making these tools more accessible and versatile for high-throughput chemical testing.

### Behavioral Screening Systems

The earliest quantitative analyses of *C. elegans* behavioral phenotypes were described over 50 years ago.^80^ A decade later, methods for measuring nematode locomotion behaviors were automated using video systems and microcomputers that were capable of tracking the movement of about 25 animals in real time at 1 Hz.^81^ Since that time, many trackers have been introduced to increase the throughput, sensitivity, and the number of features extracted from recordings of nematode behavior.^82–87^ Although many of these systems still operate at relatively low throughput, the recently described loopbio platform achieves a scale suitable for screening behavioral responses to environmental contaminants.^44^

The loopbio behavioral phenotyping platform uses a modular array of six 12-megapixel cameras that can record nematode behavior across a standard multiwell microplate and extract thousands of phenotypic features from freely moving nematodes within each well.^44^ Its modular design enables scalable throughput, allowing researchers to tailor the platform to their needs, including achieving throughput levels far beyond those of LPFB HTS systems. The key innovation of this system is its capacity to record behavior across a wide area at high spatial and temporal resolutions, enabling the identification and tracking of distinct nematode body regions across the entire microplate simultaneously. Once raw positional data are collected, computational ethology techniques can be applied to analyze specific behavioral endpoints and detect differences between chemically exposed individuals and controls.^42,43,88^

Behavioral screening systems offer a mix of advantages and drawbacks. For example, a key advantage of behavioral HTS platforms is that they are better suited to reveal neurotoxic effects of environmental chemicals compared with methods that measure developmental or lethality endpoints. Behavioral responses are often more sensitive indicators for some forms of toxicity, with several studies reporting measurable effects at concentrations orders of magnitude below those causing lethality.^89,90^ On the other hand, the recent advances in the throughput of behavioral HTS platforms require a significant amount of computational effort to extract phenotypes from large numbers of chemicals, including significant data handling and storage costs.

### Large-Particle Flow-Based (LPFB) Systems

The Complex Object Parametric Analyzer and Sorter FlowPilot (COPAS FP) system is a large-particle flow analysis platform designed to rapidly measure and sort nematodes based on length, optical density, and fluorescence.^45^ This platform enables researchers to quantify growth patterns and assess overall health indicators in animals exposed to various chemicals. Laser-based detection captures individual nematode characteristics in real time as they pass through a precisely calibrated flow cell, providing insights into size variation, optical density shifts, and fluorescence intensity changes. These parameters together offer a detailed profile of nematode physiology under the experimental conditions. The system’s high-throughput capacity allows for the rapid analysis of large chemical libraries, accelerating data acquisition and improving statistical power to support robust toxicological conclusions.^21,47,91^

In toxicology studies, the COPAS FP system enables investigations of how chemical exposures affect nematode health by quantifying growth dynamics, reproductive fitness, and stress response markers.^8,21,46–49,51,92–97^ Its versatility allows adaptation to diverse experimental paradigms, from assessing environmental pollutants to testing drug candidates or exploring fundamental biological processes. Notably, several groups have developed chemical screening workflows that are useful for toxicological research. For example, one group used the COPAS FP system to measure developmental delays in response to a large panel of environmental toxicants tested across a range of seven or more doses.^47,98^ Using a similar assay paradigm, massively scaled chemical screening strategies were used to assess toxicant exposures across genetically diverse strains.^46,48,49,99^ More recently, a multigenerational platform was developed using a nonreplicative food source, composed of nonliving *E. coli* bacterial ghosts^100^ to minimize microbial metabolism and improve assessments of toxicological endpoints over multiple generations.^52^ Overall, the COPAS FP system represents a valuable tool for nematode-based research, offering efficient, precise, and adaptable methods to evaluate chemical effects and advance toxicology, drug discovery, and basic biology.

### Image-Based Systems

Image-based HTS platforms represent a major advancement in nematode toxicological research, particularly for studies involving *C. elegans* and related nematode species. These systems measure many of the same chemical response endpoints as LPFB systems but offer several advantages: they are cheaper and simpler as they do not require precision fluidic components used in LPFB systems, the throughput is an order of magnitude higher than LPFB systems, they provide visual confirmation of results, and they generate image data sets that can be reanalyzed to extract additional morphological or fluorescence-derived phenotypes. However, a primary limitation of image-based systems is that segmenting animals from images backgrounds and extracting phenotypes is difficult to automate and prone to error.^101^ To address this difficulty, several software tools have been developed, including CellProfiler WormToolbox,^101,102^ WormSizer,^103^ WormScan,^104^ QuantWorm,^105^ and WorMachine.^106^ More recently, tools that extend and enhance the functionality of these platforms have emerged, such as wrmXpress^107^ and easyXpress,^55^ which facilitate more robust and reproducible downstream analyses.

One image-based system that is particularly well suited for assessing toxicological endpoints at scale uses an inverted epifluorescence microscope with an automated stage to acquire images, which are then processed using CellProfiler and easyXpress (Figure 1). This platform supports detailed examination across formats such as multiwell plates and microscope slides, enabling efficient genetic and chemical screening. A key advantage of this image-based HTS is its ability to automatically capture high-resolution data across large sample sizes with minimal human input, an essential feature for evaluating both morphological and developmental endpoints. The platform’s flexibility to incorporate multiple nematode strains in a single experiment also enables broad comparative studies, making it ideal to investigate the genetic underpinnings of chemical responses that vary among individuals in a population.^108^

**Figure 1:**
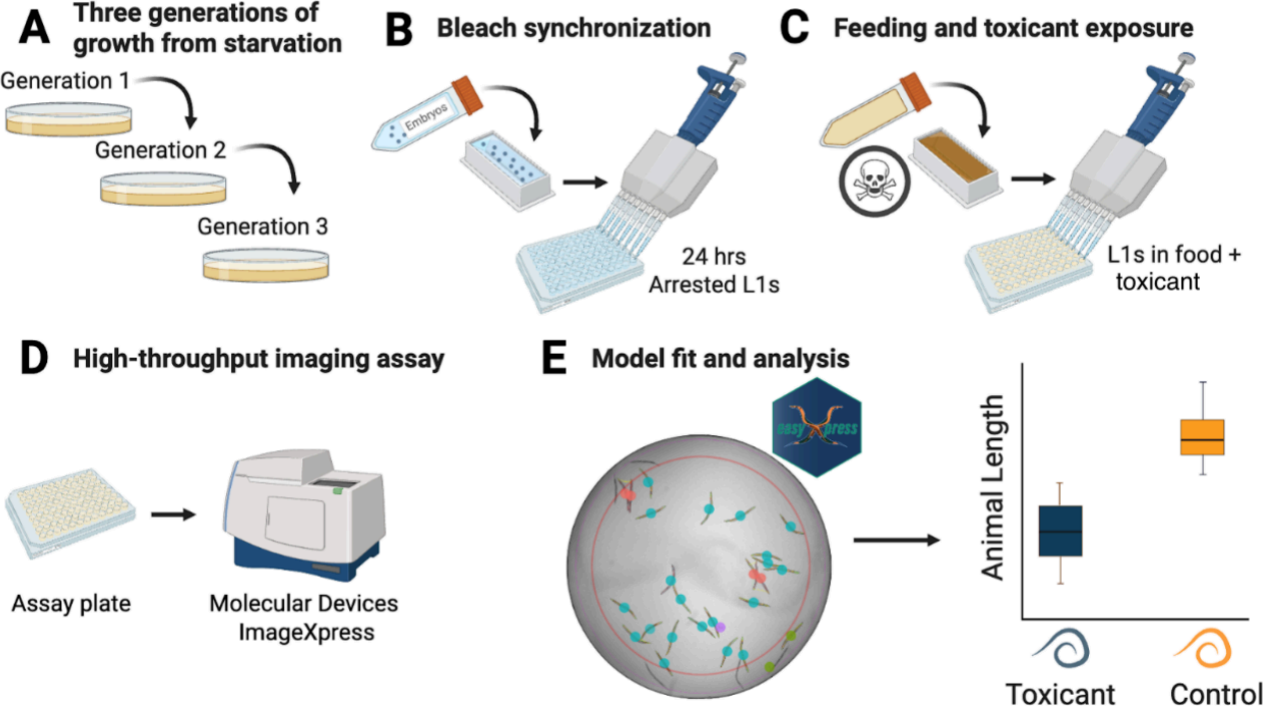
*Caenorhabditis elegans* imaging-based system and workflow to study the quantitative effects of toxicants on larval development.

The integration of high-throughput imaging, precise environmental control, and robust data analysis underscores the system’s potential to transform nematode-based screening. For example, in a recent study, researchers used this image-based HTS platform to perform dose–response analyses on 23 toxicants using eight *C. elegans* strains at high replication.^53^ By exposing first larval stage animals to toxicants and modeling strain-specific dose–response curves, they demonstrated that natural genetic variation plays a key role in determining the toxicant susceptibility. Leveraging standing natural genetic variation in *C. elegans* represents a powerful strategy for high-throughput risk assessments in translational toxicology. Overall, the imaging-based HTS platforms offer scale and flexibility that surpass all other available systems with only small limitations in data processing and storage.

## Toxicity Variation within Nematodes and across Other Toxicology Models

### Intraspecific Variation among *C. elegans* Strains and Related Species

Before comparing *C. elegans* testing platforms to those of other commonly tested species, it is essential to highlight the importance of intraspecific variation within *C. elegans*. Widmayer et al. (2022) analyzed dose–response relationships for 23 environmental toxicants across eight *C. elegans* strains representing intraspecific genomic diversity.^53^ They observed substantial variation in toxicity estimates among strains, demonstrating that genetic background is a key driver of chemical sensitivity within species (Figure 2). Importantly, for every toxicant tested, at least one wild *C. elegans* strain showed significantly different sensitivities compared to the standard N2 laboratory strain. A similar study found chemical class-specific and strain-dependent variation in anthelmintic drug responses.^54^ Further studies have explored strain-specific responses to metals such as cadmium, nickel, and arsenic, identifying genetic differences at the gene level as contributors to toxicity variation.^49,109,110^ In a separate non-HTS study, Heaton et al. (2022) found that wild strains were more sensitive to copper than N2 and noted that N2’s sensitivity declined over time with standard laboratory culturing.^111^ These studies illustrate how intraspecific testing in *C. elegans* can reveal the functional genetic variation underlying mechanistic differences in chemical sensitivity, an essential consideration for improving the utility of HTS systems for predictive toxicology.

**Figure 2:**
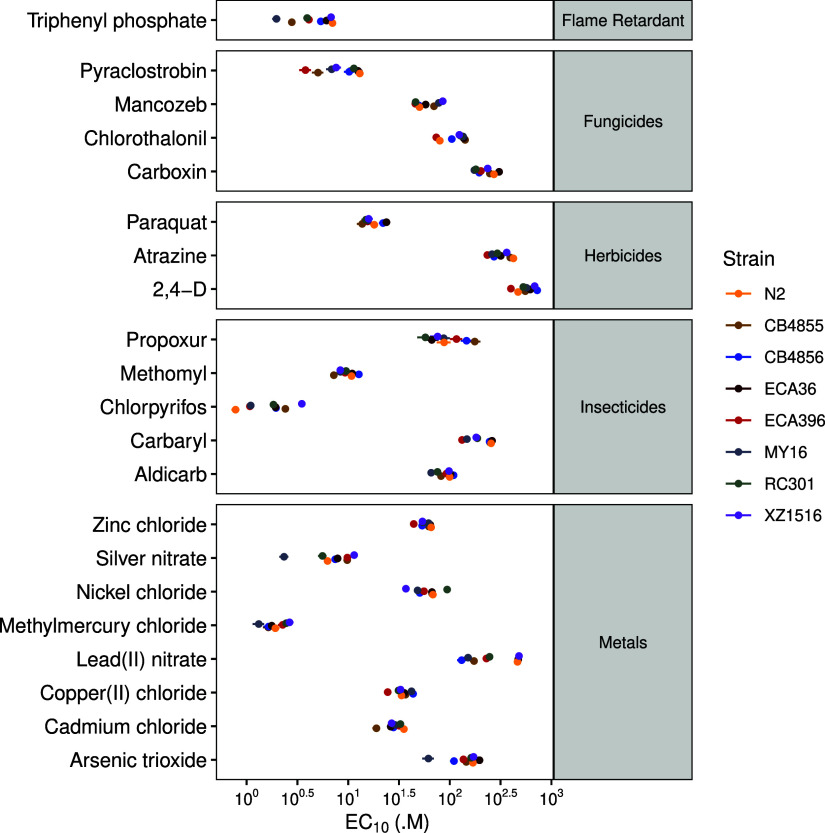
Natural variation in toxicant responses among *C. elegans* strains. EC_10_ estimates from a log–logistic model (*x*-axis) are displayed for each strain (color) and toxicant (*y*-axis). Standard errors for each EC_10_ estimate are shown as lines extending through each point. Toxicants are grouped into broad classes labeled on the right (Modified from Widmayer et al., 2022).

Beyond *C. elegans* strains, several studies have demonstrated significant differences in chemical sensitivity among nematode species within the Rhabditidae family. For example, Heaton et al. (2021) and Boyd and Williams (2003) found that *C. elegans* exhibited greater metal tolerance than *Pristionchus pacificus*, a closely related nematode.^112,113^ These findings highlight the value of comparative analyses across nematode species, especially given that *C. elegans*, *C. briggsae*, *C. tropicalis*, *Oscheius tipulae*, and *P. pacificus* can often be cultured and tested under similar conditions. However, it is important to recognize that these species have distinct temperature optima and physiological requirements.^114–116^ Rearing non-*C. elegans* species under standardized *Caenorhabditis elegans*-like conditions can introduce stress, potentially confounding toxicological outcomes. Therefore, careful attention to species-specific developmental rates, environmental preferences, and stress responses is essential to ensure valid comparisons and reliable interpretation of toxicity data across species.

Taken together, these studies highlight the importance of careful strain and species selection and management in nematode HTS. Prolonged laboratory culturing can result in artificial selection or genetic drift, potentially leading to reduced sensitivity or generalized chemical tolerance.^111,117^ To mitigate this issue, strains should be regularly refreshed from cryopreserved stocks, a straightforward process in nematodes that is not possible with most other model species. Additionally, incorporating multiple strains or species, including wild strains, into routine HTS protocols is essential for capturing realistic intra- and interspecific variation in chemical sensitivity.

### Comparison of *C. elegans* HTS Platforms

Given the general advantages of the *C. elegans* model for HTS of chemicals and the different capabilities of image-based and LPFB HTS systems, we sought to directly compare toxicological endpoint values estimated by each system. Both image-based and LPFB systems primarily report growth measurements, but we identified only 11 overlapping chemicals across published data sets.^47,53^ Within this small sample and excluding carbaryl as an outlier, growth sensitivity estimates were generally consistent across platforms (Figure 3a), suggesting that both systems capture similar toxicity profiles (*R* ^2^ = 0.55; *n* = 10). Differences in the HTS platforms could reduce interplatform consistency. For example, the LPFB system infers growth from extinction values rather than direct size measurements,^47^ which could make it more sensitive to toxicant-induced changes in internal opacity than image-based systems. However, to evaluate cross-platform performance more thoroughly, broader chemical testing, spanning diverse classes and mechanisms of action, are needed. Future research should focus on systematically expanding chemical overlap and increasing the diversity of compounds tested to strengthen platform comparability and confidence in *C. elegans*-based HTS. Importantly, the image-based platforms offer practical advantages over LPFB systems, including reduced cost, simpler instrumentation, and access to rich morphological and fluorescence-derived phenotypes. If future studies confirm that image-based endpoints correlate better with ecological and human health surrogates, such advantages could drive a broader shift toward image-based HTS.

**Figure 3:**
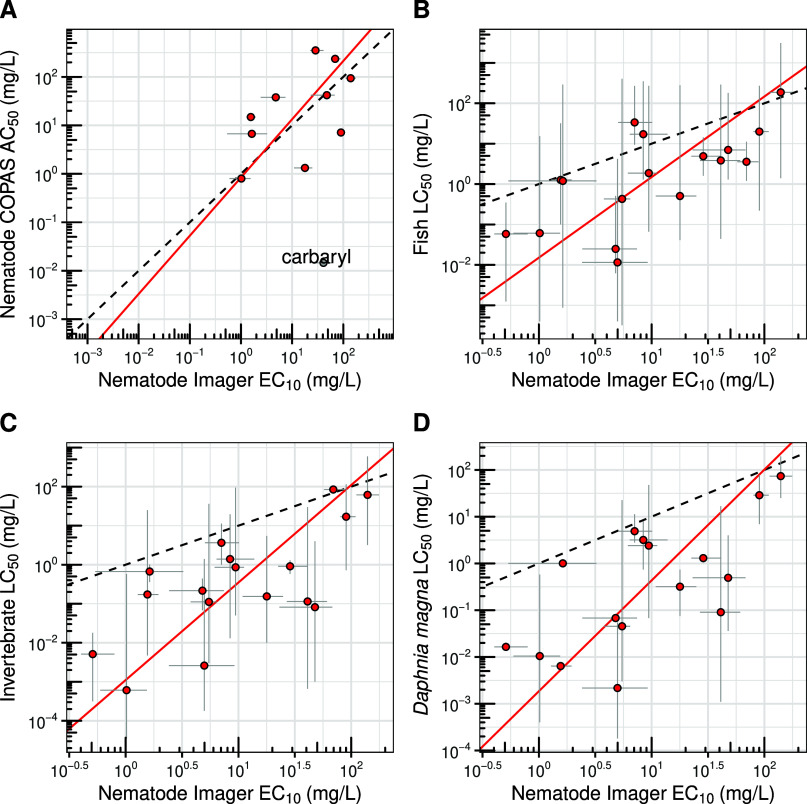
Comparison of *C. elegans* toxicity data across platforms and taxonomic groups. All comparisons are based on orthogonal regression. In each plot, the dashed black line represents unity, and the red solid line represents the orthogonal regression. Red points indicate the geometric mean, and gray lines show the full range of individual observations. (A) *C. elegans* imaging-based EC_10_ (*x*-axis) vs LPFB AC_50_ (*y*-axis) (slope = 1.202, *y*-intercept = −0.076, *R* ^2^ = 0.55, *n* = 10 chemicals). Red points were included in the regression, and the gray point (carbaryl) was excluded as an outlier. (B) *C. elegans* imaging-based EC_10_ (*x*-axis) vs 96 h acute fish LC_50_ (*y*-axis) (slope = 1.99, *y*-intercept = −1.82, *R* ^2^ = 0.75, *n* = 17 chemicals). (C) *C. elegans* imaging-based EC_10_ (*x*-axis) vs 48 h acute invertebrate LC_50_ for mortality (*y*-axis) (slope = 2.5, *y-*intercept = −2.96 *R* ^2^ = 0.82, *n* = 17 chemicals). (D) *C. elegans* imaging-based EC_10_ (*x*-axis) vs 48 h acute *Daphnia magna* LC_50_ for mortality (*y*-axis) (slope = 2.37, *y*-intercept = −2.73, *R* ^2^ = 0.79, *n* = 16). EC_10_ values were used for *C. elegans* because more chemicals had EC_10_ values for growth compared to EC_50_ values, allowing for a larger sample size.

### Comparing *C. elegans* HTS Data to Ecological Surrogates

Environmental toxicology studies consistently demonstrate robust cross-species correlations in chemical sensitivity, even among distantly related taxa.^118^ To evaluate the predictive value of nematode HTS platforms within this broader context, we compared *C. elegans* HTS data to toxicity data from a diverse set of aquatic models, including fish, aquatic invertebrates, and algae, which represent the three major taxonomic groups commonly used in chemical safety testing for aquatic risk assessment (Table 2).^33^

**Table 2:** Regression Relationships between *C. elegans* HTS Platforms and Common Ecological or Human Health Surrogates^a^

species group 1	species group 2	OR slope (95% CI)	OR intercept (95% CI)	OR *R* ^2^	slope	intercept	*n*
nematode (EC_10_)	nematode (AC_50_)	4.82 (−16.5 to 26.14)	–4.7 (−32.24 to 22.85)	0.495	0.361	0.583	11
nematode (EC_10_)	algae (EC_50_)	11.58 (−49.99 to 73.14)	–11.38 (−67.95 to 45.19)	0.526	0.187	–0.229	12
nematode (EC_10_)	fish (LC_50_)	1.99 (0.81 to 3.18)	–1.82 (−3.25 to −0.39)	0.75	1.091	–0.922	17
nematode (EC_10_)	invertebrate (EC_50_)	2.5 (1.46 to 3.54)	–2.96 (−4.36 to −1.56)	0.815	1.372	–1.834	17
nematode (EC_10_)	rat (LD_50_)	5.01 (−56.52 to 66.53)	–3.15 (−79.39 to 73.08)	0.292	0.153	2.46	12
nematode (EC_10_)	zebrafish (AC_50_)	1.14 (−1.71 to 3.98)	–0.67 (−3.77 to 2.44)	0.249	0.255	0.132	8
nematode (AC_50_)	algae (EC_50_)	2.25 (−5.68 to 10.18)	–2.67 (−13.91 to 8.56)	0.294	0.271	0.014	161
nematode (AC_50_)	fish (LC_50_)	9 (−3.17 to 21.17)	–11.38 (−27.97 to 5.2)	0.369	0.127	0.559	504
nematode (AC_50_)	invertebrate (EC_50_)	11.49 (−3.14 to 26.12)	–15.07 (−35.06 to 4.91)	0.449	0.139	0.182	413
nematode (AC_50_)	rat (LD_50_)	0.06 (−0.07 to 0.19)	3.05 (2.86 to 3.24)	0.31	0.028	3.094	550
nematode (AC_50_)	zebrafish (AC_50_)	0.91 (−0.2 to 2.01)	–0.9 (−2.47 to 0.67)	0.167	0.164	0.105	401
fish (LC_50_)	algae (EC_50_)	1.08 (0.61 to 1.54)	–0.13 (−0.5 to 0.23)	0.343	0.351	0.206	153
fish (LC_50_)	invertebrate (EC_50_)	1.13 (1.04 to 1.21)	–0.36 (−0.48 to −0.23)	0.803	0.882	–0.212	384
fish (LC_50_)	rat (LD_50_)	0.15 (0.08 to 0.22)	2.94 (2.84 to 3.04)	0.615	0.115	2.966	385
fish (LC_50_)	zebrafish (AC_50_)	0.87 (0.67 to 1.08)	0.39 (0.29 to 0.48)	0.595	0.545	0.34	253
invertebrate (EC_50_)	algae (EC_50_)	0.65 (−0.09 to 1.4)	0.2 (−0.21 to 0.6)	0.174	0.149	0.347	140
invertebrate (EC_50_)	rat (LD_50_)	0.21 (0.14 to 0.28)	2.91 (2.82 to 3.01)	0.699	0.169	2.93	310
invertebrate (EC_50_)	zebrafish (AC_50_)	0.32 (0.12 to 0.51)	0.37 (0.25 to 0.5)	0.419	0.179	0.316	200
algae (EC_50_)	rat (LD_50_)	–0.05 (−0.16 to 0.06)	3.04 (2.93 to 3.15)	0.645	–0.036	3.036	123
algae (EC_50_)	zebrafish (AC_50_)	0.33 (−0.07 to 0.73)	0.25 (0.04 to 0.45)	0.278	0.135	0.267	91
rat (LD_50_)	zebrafish (AC_50_)	7.8 (−12.62 to 28.22)	–23.43 (−85.7 to 38.84)	0.278	0.096	–0.017	241

a “Nematode (EC_1__0_)” values were derived from an image-based platform^53^ measuring larval growth using microscopy and automated image analysis. “Nematode (AC_5__0_)” values came from a LPFB COPAS system^47^ quantifying optical density and extinction as proxies for animal size after chemical exposure. Aquatic species data, including fish (LC_5__0_), invertebrate (EC_5__0_) for immobility or mortality, and algae (EC_5__0_) for cell counts, were taken from the EnviroTox database.^119^ Rat (LD_5__0_) values were obtained from the National Toxicology Program’s Integrated Chemical Environment (ICE) database.^121^ Zebrafish (AC_5__0_) values refer to high-throughput embryo assays from ToxCast Phase I–II.^122^ The columns “OR Slope”, “OR Intercept”, and “OR *R* ^2^” report orthogonal regression estimates. The “Slope” and “Intercept” columns report ordinary least squares regression coefficients.

We first examined the LPFB COPAS data set from Boyd et al.,^47^ which includes AC_5__0_ values (the assay concentration at which 50% of the maximal response is observed) for 959 chemicals from US EPA’s ToxCast Phase I and II libraries. We compared these values to aquatic toxicity data from the EnviroTox Database^119^ using orthogonal regression, which accounts for uncertainty in both data sets.^120^ Specifically, we compared LPFB COPAS AC_5__0_ data to fish 96 h LC_5__0_ data, aquatic invertebrate 48 h EC_5__0_ data for immobility or mortality, and algal 96 h EC_5__0_ data for cell counts. Because only overlapping chemicals can be analyzed, sample sizes varied by taxonomic group. We found relatively weak relationships between *C. elegans* and aquatic surrogate species, with *R* ^2^ values of 0.37 for fish (*n* = 504), 0.45 for aquatic invertebrates (*n* = 413), and 0.29 for algae (*n* = 161). By contrast, aquatic species exhibited strong intercorrelations with one another, including an *R* ^2^ of 0.80 between fish and aquatic invertebrates (*n* = 384), highlighting greater internal consistency within aquatic models. The weaker relationships with *C. elegans* might reflect limitations in the LPFB COPAS assay design, such as fixed test concentrations and large spacing factors, which increase the uncertainty in AC_5__0_ estimates. Additionally, the data set encompassed a broad array of chemical classes (*e.g*., metals, pesticides, industrial compounds), which could further obscure specific cross-species trends.

In contrast, the *C. elegans* image-based HTS data set from Widmayer et al. (2022), which reported EC_1__0_ values for 23 environmental toxicants, showed stronger alignment with established ecological surrogates. The relationships were strongest for fish (*R* ^2^ = 0.75; *n* = 17; Figure 3B) and aquatic invertebrates (*R* ^2^ = 0.82; *n* = 17; Figure 3C). The correlation with algae was weaker (*R* ^2^ = 0.53; *n* = 12) but still higher than the LPFB-to-algae comparison (*R* ^2^ = 0.29; *n* = 161). At the species level, *C. elegans* image-based data aligned most closely with the aquatic invertebrate model *Daphnia magna* (water flea; *R* ^2^ = 0.79; Figure 3D) and with the fish models *Oncorhynchus mykiss* (rainbow trout; *R* ^2^ = 0.78) and *Pimephales promelas* (fathead minnow; *R* ^2^ = 0.75). Together, these results suggest that experimental methods and assay design, not the organism *per se*, govern cross-taxon concordance, with image-based data tracking aquatic outcomes more closely than LPFB outputs.

Preliminary range-finding experiments and increased replication (2-fold) likely contribute to the improved cross-species predictivity of image-based HTS data relative to LPFB data.^47,53^ The range-finding experiments used for the image-based assays made it possible to define complete dose–response relationships for chemicals and avoid extrapolation of endpoints. Moreover, the block design and greater replication facilitated the removal of outliers and block effects using regression analysis.^53^ Importantly, greater replication is possible with image-based systems without additional labor because automated imaging decreases the time required to process a single plate by an order of magnitude relative to LPFB systems (∼2 min vs ∼20 min). LPFB systems are necessarily slower because they must first aspirate samples from wells then pass them through a flow cell to measure the well content.^45^ Moreover, LPFB systems often use fewer treatments per plate because wash wells are included between treatments. This approach can reduce the number of usable wells relative to image-based systems by 20–25%, depending on the experimental design used.^47^ Additional equipment such as the COPAS LP sampler can be used to remove the need for wash wells, but this increases the cost of the LPFB system and does not significantly reduce sampling time.^99^

### Comparisons of *C. elegans* HTS Data to Human Health Surrogates

Given the concordance with aquatic models, we next asked how well *C. elegans* HTS endpoints align with established human health surrogates. Overall, concordance was lower than that for the aquatic ecological surrogates (Table 2). Correlations with rat acute oral toxicity sourced from the National Toxicology Program’s Integrated Chemical Environment (ICE) database^121^ were modest (LD_5__0_; *R* ^2^ = 0.29*, n* = 12), and so was concordance with a zebrafish embryo HTS lethality/malformation endpoint^122^ (AC_5__0_; *R* ^2^ = 0.25; *n* = 8). The LPFB COPAS data set also showed similarly weak correlations but with much larger chemical overlaps (rats *R* ^2^ = 0.31, *n* = 550; zebrafish *R* ^2^ = 0.17, *n* = 401). We also attempted to align image-based *C. elegans* results with US EPA ToxCast HTS assays, but after filtering out assays with poor dose–response fits or unbounded endpoints,^123^ the chemical overlap was insufficient for a robust analysis. These findings underscore a practical barrier: the lack of overlap across data sets. They also highlight the need for coordinated testing to evaluate the translational potential of the *C. elegans* HTS.

Given the sparse overlap among data sets, especially for promising image-based data, we recommend assembling shared reference panels of compounds for future *C. elegans* HTS. Although defining a definitive list is beyond the scope of this review, compounds within the panels should meet five criteria: (1) characterized human and environmental effects, (2) known physicochemical properties, (3) coverage of the relevant chemical space (e.g., pesticides, pharmaceuticals, industrial chemicals), (4) ready commercial availability and HTS compatibility, and (5) a balanced set of chemicals established to be toxic and nontoxic in other surrogates, enabling estimation of true-positive and true-negative prediction rates. Using shared panels would improve cross-study comparability, enable method benchmarking against common reference chemicals, and provide internal assay benchmarks to support quality control and lab-to-lab standardization. The National Toxicology Program’s 87-compound developmental neurotoxicity panel is an excellent example, although it includes just five nontoxic control compounds.^124^ Researchers applied a battery of *in vivo* and alternative animal model assays (zebrafish and planarian) to characterize neurotoxicity across the chemical panel.^125,126^ Among 28 chemicals with mammalian toxicity data in the EPA Toxicity Reference Database, 96% were bioactive in either zebrafish or planarian systems, supporting the predictivity of these models for mammalian toxicity and illustrating the broader importance of shared reference panels.^127^ Another example is the OECD-recognized list of positive and negative compounds for evaluating the viability of alternative testing systems (*e.g.*, NAMs, *in vitro*) for detecting neurotoxicity.^128^

Beyond improving data set overlap, expanding coordinated *C. elegans* HTS efforts would also strengthen their use within the AOP framework. Because AOPs emphasize conserved molecular initiating events (MIEs), downstream key events (KE), and key event relationships (KERs),^62,129,130^ systematic HTS profiling in *C. elegans* will help identify cases where nematode responses track human-relevant pathway perturbations, strengthening their use for predicting mammalian toxicity. For example, a recent RNAi screen using the LPFB HTS platform identified nucleotide excision repair and TGF-β signaling as KEs in high-density polyethylene (HDPE) microplastic toxicity.^131^ These pathways were subsequently validated in zebrafish and bioinformatic analysis of the Comparative Toxicogenomics Database (CTD)^132^ linked them to human disease.^131^ This example clearly illustrates how *C. elegans* HTS efforts can generate mechanistic evidence with relevance to human risk assessment.

## Findings, Advantages, and Uncertainties

### Robust Performance of Image-Based HTS

We found a strong concordance between *C. elegans* and established ecotoxicological model responses, particularly in assays using image-based HTS (Table 2). In many cases, growth and developmental endpoints in *C. elegans* track closely with apical outcomes observed in fish and invertebrate toxicity tests, supporting its relevance as a predictive model. The sensitivity and resolution offered by automated microscopy platforms enable reliable quantification of sublethal effects at scale, making this approach a strong fit for broad chemical screening and comparative hazard assessment.

### Distinct Sensitivity Patterns in *C. elegans*

Despite these correlations, *C. elegans* often shows reduced sensitivity at lower concentrations compared to aquatic species such as *Daphnia magna* (Figure 3B–D). This difference is somewhat unexpected given the stronger congruence typically observed across traditional aquatic models.^118^ We suggest that these differences are more likely caused by biological differences than by methodological limitations. Nematodes are adapted to harsh terrestrial environments and possess robust external barriers, such as a collagen-rich cuticle supported by over 150 collagen genes,^133^ that can reduce chemical absorption and bioavailability. This observation is consistent with the hypothesis that *C. elegans* predominantly models oral toxicity,^63^ whereas toxicity in *D. magna* arises through both ingestion and absorption.^134,135^ Ultimately, direct measurement of internal chemical concentrations in nematodes would help clarify the contribution of the uptake to these patterns.

### Unique Contributions of *C. elegans* to HTS

Compared to other small animal models such as zebrafish and *Daphnia*, *C. elegans* and related nematodes offer distinct advantages that make them highly attractive for the standardized HTS of chemicals. Their small size, ease of culture, genetic tractability, and compatibility with automated platforms enable scalable, full life-cycle testing within as little as 3 days.^136^ By contrast, zebrafish require approximately 90–120 days to reach reproductive maturity, and *D. magna* take 21–28 days to complete a full life cycle. Existing OECD Test Guidelines typically capture only part of this life cycle: 96 h for zebrafish embryos (TG 236), 48 h for acute *Daphnia* immobilization (TG 202), and 21 days for reproduction endpoints (TG 211). Even compared to new *in vitro* systems like the RTgill-W1 cell line assay (TG 249), which assesses acute cytotoxicity in just 24 h, nematodes uniquely combine whole-organism complexity across life stages with HTS feasibility, bridging the gap between simple cell-based assays and vertebrate-focused tests. In a properly equipped laboratory, range-finding and dose–response assays for multiple developmental and behavioral endpoints can be completed across many toxicant-strain combinations in a single week, without the added cost and time associated with Institutional Animal Care and Use Committee (IACUC) oversight. These features position *C. elegans* as an unparalleled system for next-generation HTS assay development.

Despite differences in sensitivity, *C. elegans* also captures a distinct and complementary spectrum of toxic effects, particularly when used alongside zebrafish and other models. For example, although zebrafish embryos might show limited sensitivity to certain neurotoxicants,^137^ the connectome of *C. elegans* is better described and has conserved neurotransmitter pathways (*e.g*., acetylcholine, dopamine, GABA, glutamate, serotonin), offering enhanced sensitivity to neuroactive compounds.^38,138^ Although current OECD guidance for developmental neurotoxicity testing is focused on mammalian *in vivo* studies (*e.g*., TG 426) and emerging *in vitro* assays coordinated through the Developmental Neurotoxicity (DNT) In-Vitro Testing Battery initiative,^128^ whole-organism new approach methodologies (NAMs) like *C. elegans* remain underexplored. Recently, novel experimental approaches are emerging that leverage the advantages of *C. elegans* to thoroughly assess the effects of environmental pollutants on the developing nervous system at both structural and functional levels using fluorescent imaging and behavioral assays.^139^ Additionally, chemically induced dopaminergic neurodegeneration has been directly linked to functional behavioral deficits in *C. elegans* using highly scalable analyses involving automated image processing and microfluidic devices.^140^ Future work can help determine whether *C. elegans* HTS can serve as a gap-filling model for detecting specific classes of neurotoxicants not reliably captured by current systems.

Furthermore, *C. elegans* offers important ecological and mechanistic diversity to toxicity testing frameworks that are otherwise dominated by aquatic vertebrates and arthropods. Nematodes are key members of the terrestrial microfauna, yet standardized terrestrial invertebrate models remain limited. Only a few OECD guidelines currently address terrestrial species, including tests for earthworms (TG 207 and 222), predatory mites (TG 226), and collembolans (TG 232). Compared to these larger or less genetically tractable species, *C. elegans* offers unique advantages, including reproducible developmental timing, transparent anatomy, and precision in quantifying sublethal endpoints such as growth, reproduction, and behavior. These features, combined with its compatibility with HTS platforms, position *C. elegans* as a powerful and complementary model for filling ecological and functional gaps in current toxicological test batteries.

Regulatory agencies have long recognized that genetic differences among individuals can influence toxicant responses and chemical risk assessment can be improved by identifying the genetic factors that drive differences in chemical susceptibility among populations.^141,142^ *C. elegans* and related nematodes are uniquely suited to discover genetic variants that influence chemical susceptibility because the *Caenorhabditis* Natural Diversity Resource (CaeNDR) provides thousands of publicly available, genetically diverse, and whole-genome-sequenced wild strains isolated from around the world.^17^ HTS experiments using these defined nematode populations have already characterized how natural genetic variation shapes responses to multiple toxicants.^46,48,49,109,143–145^ Although genetically diverse mammalian populations, such as the Collaborative Cross^146^ and Diversity Outbred^147^ mouse populations, have also been used for these purposes,^148–150^ mammalian models are inherently low-throughput and expensive, making them less suited for large-scale experimentation. Genetically diverse populations of zebrafish have also enabled researchers to measure and map variation underlying chemical responses,^151,152^ but the capacity to perform HTS across all life history stages, the low cost, and the unrivaled genetic tractability of *C. elegans* make it particularly well suited for these studies.

### Limitations of Current Platforms

Several limitations of current *C. elegans* HTS platforms can be resolved to support broader regulatory uptake or use for chemical prioritization. For example, most *C. elegans* HTS platforms require feeding nematodes throughout the exposure period, as continuous access to food is essential for normal development, growth, and reproduction. However, the use of live bacterial food sources (typically *E. coli*) can introduce confounding variables, including endotoxin production^153,154^ and metabolic degradation of test compounds, both of which could alter exposure profiles and either exaggerate or diminish toxic effect measurements.^155^ Although axenic or chemically defined media systems have been developed to eliminate these complications,^156^ most do not support normal growth of *C. elegans* and should not be adopted for regulatory toxicology.^157–159^ In contrast, *C. elegans* Habitation Medium (CeHM), made of 80% *C. elegans* Habitation Reagent (CeHR) and 20% nonfat cows’ milk, does support developmental rates similar to feeding *E. coli* on agar plates and has been used successfully in recent toxicology studies.^160–163^ Alternatively, chemical- or heat-killed *E. coli* might address concerns about bacterial effects on chemicals.^164–166^ More recently, experimental methods to reduce the influence of live bacterial food sources in toxicology include the use of nonreplicative bacterial ghosts.^52^

Temperature, humidity, sample volumes, and chemical absorption by culturing materials can all influence the performance of the HTS platforms reviewed here. For example, temperature and humidity fluctuations can cause variable levels of media evaporation across the wells of microplates used with image-based and LPFB platforms,^167^ although several approaches, such as controlled incubators, humidity chambers, and sealing films, can mitigate these effects.^46,168^ Another potential limitation is chemical absorption by the culture materials. For example, PDMS commonly used in microfluidic devices can absorb some chemicals, lowering their effective concentrations and altering experimental results.^169^ In these cases, devices made of alternative materials may be superior.^170,171^ Overall, careful consideration of both media evaporation and material-dependent absorption is essential to maintain consistent exposure concentrations in HTS experiments.

*C. elegans* and related nematodes develop quickly to adulthood in favorable conditions but can enter a longer-lived dispersal stage (called dauer) when environments become unfavorable.^172^ Stressors like nutrient limitation or particular toxicants can induce dauer formation.^63^ This stage poses two challenges for toxicant HTS. First, dauer larvae have thick cuticles, do not feed, and stop growing,^172^ which can interfere with the assessment of growth effects for some chemicals. Second, the presence of dauers in an experimental population can increase the expression of stress response genes in nondauer larvae in the same population,^173,174^ potentially skewing toxicant responses. Consequently, researchers should ensure adequate nutrition before and during experimentation, with the exception of deliberate posthatching larval arrest used for developmental synchronization.^175^

Some *C. elegans* HTS studies have bypassed traditional range-finding experiments designed to identify the concentration range over which chemicals elicit maximal biological responses. Instead, these studies often applied a uniform set of widely spaced concentrations across all compounds. Although this approach is highly practical and aligns with strategies used in other HTS efforts such as EPA’s ToxCast and zebrafish assays,^122,176^ it risks missing important effects or producing inaccurate potency estimates, especially when toxic responses fall outside the tested concentration range. To improve endpoint estimation, more tailored dose-setting strategies are needed, similar to those used in standardized test guidelines. Notably, Widmayer et al. (2022) and Shaver et al. (2023) employed efficiently designed preliminary screens to guide concentration selection and improve EC_5__0_ estimates using an image-based system.^53,54^ Their approach used one of two methods per chemical. If pre-existing toxicity data were available, they applied a 2-fold dilution series centered on the estimated EC_5__0_. If not, they used a dilution series with a maximum concentration near the solubility limit. Both methods tested 12 concentrations with minimal replication, enabling efficient screening of many chemicals per microplate. Experiments were iterated as needed until an appropriate response range was captured.

Although *C. elegans* HTS platforms show promising alignment with ecotoxicological models, their relevance for human health prediction remains uncertain, in part because of limited compound overlap across data sets. In our case, only a small number of chemicals were shared between the image-based *C. elegans* data set and mammalian toxicity studies or ToxCast assays after quality filtering. Consequently, the observed relationships with rat LD_5__0_ values and zebrafish embryo assays were weak to moderate and not robust enough to support confident interpretation. For rodents, this inconsistency could be partly due to differences in toxicokinetics, as shown by Wittkowski et al. (2019), who demonstrated that normalizing to internal concentrations substantially improved concordance between *C. elegans* and rat toxicity rankings,^177^ presenting a means to address this issue in future research.

Importantly, cross-species concordance is not expected for all chemicals, particularly when adverse outcomes depend on MIEs involving targets or pathways that differ across taxa. For example, neonicotinoids, a class of insecticides that function as nicotinic acetylcholine receptor (nAChR) agonists, bind more strongly to insect nAChRs than to those of vertebrates,^178^ and they are known to be orders of magnitude more toxic to insects than nematodes^179^ or vertebrates.^180^ A similar principle applies to pyrethroids, a widely used class of insecticides that produce adverse outcomes via activation and prolonged opening of voltage-gated sodium channels (VGSCs).^181^ *C. elegans* does not have VGSCs^182^ and is relatively insensitive to these chemicals.^183^ Fortunately, the AOP framework helps to contextualize these exceptions and identify the chemical classes for which *C. elegans* can offer predictive mechanistic insights.

## Toward Formalized Guideline Testing

*C. elegans* and related nematodes have strong potential as a nonvertebrate NAM for chemical safety assessment.^63,163^ Although HTS-compatible assays are not typically developed directly into OECD Test Guidelines, they are increasingly valuable for screening and prioritization in both industrial product development and regulatory environmental risk assessment. In this context, nematodes offer a complementary whole-organism alternative to existing tools, combining scalability, life cycle speed, and ecological relevance to support early tier decision making. Standardizing multiwell-based and HTS-compatible nematode assays would therefore enhance chemical prioritization pipelines while also laying the groundwork for possible future regulatory adoption.

Developing a new OECD Test Guideline remains a multiyear process requiring broad consensus and regulatory confidence. For NAMs such as *C. elegans*-based systems, several steps are essential: (1) establish harmonized protocols defining critical parameters such as exposure duration, life stage, feeding regimen, and test media; (2) demonstrate biological relevance and applicability across diverse chemical classes and modes of action; (3) conduct interlaboratory trials to establish reproducibility and transferability; and (4) validate core endpoints—such as growth, development, and behavior—as robust, quantifiable, and meaningful for hazard and risk assessment.

The recent approval of NAM-based methods such as the RTgill-W1 cell line assay (TG 249) illustrates the growing openness to alternative systems, provided they are supported by rigorous science and collaboration. Although *C. elegans* HTS likely is the most impactful in screening and prioritization, building consensus and demonstrating reliability could eventually position nematode assays for formal guideline consideration, especially in life-cycle and developmental neurotoxicity testing.

## Future Directions

To fully realize the potential of *C. elegans* in high-throughput toxicity testing, several key steps are needed (Table 3). As explained here, *C. elegans* platforms, especially image-based systems, offer strong comparative value, practical advantages, and complementary sensitivity profiles, but they require broader validation, coordinated efforts, and methodological expansion.

**Table 3:** Summary of Research Recommendations for Nematode HTS Development for Environmental Toxicology

HTS recommendations	rationale
expand chemical testing across HTS platforms	Broader chemical coverage across diverse modes of action is essential to validate cross-platform consistency, improve comparisons to vertebrate and invertebrate models, and identify where *C. elegans* HTS performs well or underperforms.
use multiple *C. elegans* strains and species	Toxicity responses vary significantly across wild and lab *C. elegans* strains and among closely related nematode species. Including ≥ 4 strains and additional species (*e.g*., *C*. *briggsae*, *P*. *pacificus*) enhances predictive power and ecological relevance.
renew test strains regularly from cryopreserved stocks	Long-term culturing of *C. elegans* can lead to laboratory adaptation and reduced chemical sensitivity. Routine renewal from cryopreserved stocks, available for both wild and lab strains, helps preserve genetic integrity and ensures consistent assay performance over time.
integrate behavioral endpoints with growth assays	Behavioral HTS platforms may capture subtle neurotoxic effects often missed by growth-based systems. Combining both approaches increases detection sensitivity and supports a broader range of toxicity mechanisms.
adopt tailored dose-setting strategies	Uniform dosing approaches risk mis-estimating potency. Using preliminary screens or solubility-informed dilution series improves EC_5__0_ estimates and maximizes data quality across compounds.
minimize microbial confounding in feeding regimens	Live bacterial food sources can metabolize test chemicals or obscure effects. Alternative approaches (*e.g*., bacterial ghosts, heat-killed *E. coli*) reduce these confounders while supporting normal development.
standardize and validate protocols for regulatory use	Harmonized protocols across exposure duration, life stage, feeding, and media are critical. Interlab trials and endpoint validation (growth, behavior, reproduction) will support guideline development.
build a collaborative path toward OECD guideline development	Advancing *C. elegans* as a new approach methodology (NAM) will require coordinated efforts to draft a Detailed Review Paper (DRP), conduct expert consultations, and submit a Draft Guideline Proposal, following OECD processes.

First, large-scale comparative data sets are critical. A coordinated effort to test a diverse set of chemicals, ideally several thousand, across *C. elegans* larval development assays and standard vertebrate, invertebrate, and *in vitro* models would allow robust cross-species characterization of chemical responses. These data could help establish *C. elegans* as a reliable first tier screening model, identifying concordance, divergence, and specific chemical classes in which the model excels or underperforms. For some chemical classes, we expect *C. elegans* responses to diverge from those of other species because of genetic differences.^59^ In these cases, transgenic strains expressing human or focal-taxon genes can help determine how specific gene functions shape responses to these chemicals. Humanized lines are already being used in studies of xenobiotic metabolism,^184^ neurodegenerative disease,^185–188^ and aging.^189^

Second, single-strain testing is insufficient for capturing the diversity of toxicological responses. As demonstrated in the *Caenorhabditis* Intervention Testing Program (CITP), outcomes can vary across strains and species.^190,191^ Depending on the research question, it could be helpful to incorporate a minimum of four genetically distinct *C. elegans* strains and, when possible, additional species (e.g., *C. briggsae*, *C. tropicalis*, and *P. pacificus*) to span broader evolutionary space, enhancing both ecological and translational relevance. In this way, chemicals can be tested across genetically diverse strains from species that diverged at least 100 million years ago.^192–194^ Though preparation of multiple strains at scale presents challenges, recent innovations in filtration and automation can address these limitations.^195^ Such HTS compatible multispecies assessments might enable community-level response estimates, similar to microcosm studies.^196^

Third, although larval development assays are currently the most scalable and validated *C. elegans* endpoint, other phenotypic assays should be further developed. Behavioral platforms, for example, leverage characterized neurobiology and can detect subtle effects not captured by morphological traits.^43,44^ A second coordinated testing initiative could evaluate concordance between *C. elegans* behavioral and developmental endpoints, as well as comparisons to vertebrate models. With growing advances in imaging and AI-based behavior analysis,^42,88,197–202^ these data-rich platforms could add unique value to toxicity prediction.

Finally, regulatory engagement will be essential. Although the OECD currently recognizes only one potentially higher-throughput *in vivo* method—the zebrafish embryo assay (TG 236)—this test still relies on a vertebrate model. A nematode-based system would provide a truly nonvertebrate, whole-organism alternative for early tier testing, aligned with 3Rs principles (Replacement, Reduction, Refinement). Coordinated community efforts to standardize and validate *C. elegans* HTS methods could establish their role as a mainstay for chemical screening and prioritization, with the potential to inform and eventually contribute to future OECD guideline development.

## Supplementary Material





## Notes

Description of collection and curation toxicological data used in analysis (PDF)
Compiled raw toxicological data set across multiple species, including chemical identifiers, strain information, exposure conditions, statistical endpoints, effect values with units, endpoint definitions, and source metadata (XLSX)
