# Supplementary material for: High-Throughput Toxicity Screening with C. elegans: Current Platforms, Key Advantages, and Future Directions
Source: Environ Sci Technol. 2026 Jan 8;60(2):1560–76. doi: 10.1021/acs.est.5c12562 (PMC12825169; doi:10.1021/acs.est.5c12562)
Supplement: Supplementary file 1 [file es5c12562_si_001.pdf]

Supporting Information

High-Throughput Toxicity Screening with *C. elegans*: Current Platforms, Key Advantages, and Future Directions

Timothy A. Crombie[a], Tobias Pamminger[b], Erik C. Andersen[c]*, Scott Glaberman[d]*

[a]Department of Biomedical Engineering and Science, Florida Institute of Technology, Melbourne, FL 32901, United States

[b]Bayer AG, CropScience Division, Monheim, 40789, Germany

[c]Department of Biology, Johns Hopkins University, Baltimore, MD 21218, United States

[d]Centre for Environmental Research and Justice, School of Biosciences, University of Birmingham, Birmingham, B15 2TT, United Kingdom

* Email: s.glaberman@bham.ac.uk
* Email: erik.andersen@gmail.com

The supporting information includes: This 1-page PDF document with one text section, and a .xlsx spreadsheet.

.XLSX spreadsheet - This file contains the harmonized raw data extracted from nine published or publicly available toxicological data sources, including Widmayer *et al.* (2022), Boyd *et al.* (2016), EPA CompTox, EnviroTox DB, NIEHS ICE, Karmaus *et al.* (2022), Klüver *et al.* (2016), Su *et al.* (2021), and US EPA ToxCast. Each row represents a single toxicant–species–endpoint record. The variables in the 'supplemental data' tab of the .XLSX file are described in the 'data dictionary' tab. The raw datasets and acquisition details, including study DOIs, and retrieval instructions, are documented in the github repository at: https://github.com/Crombie-Lab/nematode-hts-toxicology
